# Kosovo women’s knowledge and awareness of human papillomavirus (HPV) infection, HPV vaccination, and its relation to cervical cancer

**DOI:** 10.1186/s12905-021-01496-x

**Published:** 2021-10-09

**Authors:** Pranvera Zejnullahu Raçi, Fitim Raçi, Teuta Hadri

**Affiliations:** 1 Department of Obstetrics and Gynaecology, Hospital and University Clinical Service of Kosovo (HUCSK), University “Hasan Prishtina”, Lagja e Spitalit n.n, 10000 Prishtina, Kosovo; 2 Department of Epidemiology, National Institute of Public Health of Kosovo, Prishina, Kosovo; 3 Department of Obstetrics and Gynaecology, Hospital and University Clinical Service of Kosovo (HUCSK), Prishina, Kosovo

**Keywords:** Human papillomavirus infection, HPV vaccination, Cervical cancer, Kosovo

## Abstract

**Background:**

The objective of this study, the first of its kind in Kosovo, is to determine the level of Kosovo women’s knowledge and awareness of HPV infection, HPV vaccination, and its relation to cervical cancer.

**Methods:**

This cross-sectional study was conducted from July to October 2018 at the Clinic of Obstetrics and Gynecology at the Hospital and University Clinical Service of Kosovo.

**Results:**

Out of 800 questionnaires distributed, 645 were completed and returned (80.6%). Only 0.5% of women were vaccinated against HPV. The majority of respondents (66.4%) had no previous knowledge of HPV (human papillomavirus). Only 27.6% of respondents were aware that HPV is responsible for cervical cancer. About two-thirds (70.1%) of respondents had never heard of the HPV vaccine, and only 24% knew that the HPV vaccine can prevent cervical cancer.

**Conclusion:**

The level of vaccination against HPV and the level of knowledge and awareness of HPV infection is extremely low. Higher education, younger age, and living in an urban area were favorable factors and correlated with awareness of HPV infection, vaccination against it, and its relation to cervical cancer. Based on this study, there is an immediate need for developing an educational program on HPV infection and the importance of HPV vaccination as a preventative measure against developing cervical cancer.

**Supplementary Information:**

The online version contains supplementary material available at 10.1186/s12905-021-01496-x.

## Background

Genital human papillomavirus (HPV) is one of the most frequent sexually transmitted infections. Although more than 200 genotypes of HPV have been identified, a group of 15 high-risk types are known as an essential factor for development of cervical cancer[1, 2] and also vulvar, vaginal, penile, and oropharyngeal cancers [3, 4].

The worldwide prevalence of HPV infection varies from 2 to 44% [5]. Several meta-analyses indicate that the most prevalent HPV types are HPV16, HPV 18, HPV 31, HPV 52, and HPV 58 [6, 7] and HPV 16 and 18 are considered an etiologically determinant agent in over 71% of cervical cancer cases [6–8]. The prevalence of specific HPV types varies geographically, taking into consideration other cofactors, mainly socio-epidemiological ones. Recently, results were also obtained for Kosovo, where the overall HPV prevalence is 13.1%, with HPV 16 as the most prevalent type [9].

In order to prevent HPV infection and thus reduce the risk of precancerous lesions and HPV-related malignancies, three types of vaccines are in use worldwide. The bivalent vaccine (Cervarix) targets HPV 16 and HPV 18. Quadrivalent Gerdasil covers HPV 16, HPV 18, HPV 6, and HPV 11, and the last vaccine to be licensed in US and Europe, mainly in high-income countries, is nonavalent Gerdasil 9, which offers protection against HPV 16, 18, 31, 33, 45, 52, 58, 6, and 11.

By 2017, 71 countries (37%) had introduced the HPV vaccine into their national immunization program for girls, and 11 countries (6%) had also done so for boys [10]. The WHO recommends vaccination of adolescents 9 to 13 years old prior to becoming sexually active and their potential exposure to HPV [11].

A study by Klug et al. showed that awareness of HPV across all populations is poor [12]. Many health-promotion programs have sought to raise awareness of HPV vaccination, but evidence for such awareness is lacking [13]. Neighboring North Macedonia introduced primary vaccination against HPV in 2009, with a 40.1% vaccination rate reported in 2013 [14].

As a small low-income country, Kosovo has not yet established any organized screening program for cervical cancer or vaccination against HPV. The prevalence of HPV among Kosovo women is approximately at the same level as in other countries in the region, and so the risk for acquiring HPV-related malignancies is very high. Therefore it is necessary to introduce application of the HPV vaccine as well as the development of related health-promotion programs.

## Methods and material

This cross-sectional study was conducted from July to October 2018 at the Clinic of Obstetrics and Gynecology at the Hospital and University Clinical Service of Kosovo. The methodology was based on questionnaires containing relevant indicators for each attribute. On average, the clinic serves about 200 hospitalized patients and provides outpatient treatment or advice to approximately 50 patients on a daily basis. This is the only tertiary clinic in Kosovo, and it provides medical services to women from across the country.

During the study, 800 structured questionnaires were distributed to women that were hospitalized or had consultations at the outpatient unit between July and October 2018. The pretested questionnaire had three parts. The first part elicited socio-epidemiological data including the age, place of living (urban or rural) level of education as well as sexual activity of the women. The second part tested women’s knowledge of HPV infection, the HPV vaccine, and its relation to cervical cancer. The third part of the questionnaire gathered information regarding women’s knowledge and the rate of cervical cancer screening (the Pap test), also in the end specifying the source of information regarding all the issues of interest in our study.

The questions were simple to answer, with only one correct response. The questionnaires were completed by respondents and then gathered at the collection point in nurses’ offices in the departments at the clinic. Out of 800 questionnaires, 645 were completed properly and returned. The remainder were not filled out correctly or were not returned for final analysis. The overall response rate was 80.6%.

For more details regarding the questions find the questionnaire attached to the manuscript.

### Statistical analyses

#### Method description

Categorical variables were described by frequencies and percentages, and continuous variables by means and standard deviations. Multivariable logistic regression was used to study the association between demographic, social, and informative factors and awareness about the cause and prevention of cervical cancer, as well as the association between demographic, social, and informative factors and understanding the cause and prevention of cervical cancer. *P* values < 0.05 were considered statistically significant. Statistical analysis was performed using SPSS, v. 26.

## Results

Out of 800 questionnaires distributed, 645 were completed correctly (80.6%). Out of this number, 295 respondents (45.7%) belong to the age group 18–25, followed by 187 (29%) in the age group 26–33. Only four (0.6%) respondents were older than 58. Regarding their place of residence, 345 (53.5%) of women lived in urban areas, and 300 (46.5%) lived in rural areas. According to level of education, the respondents were divided into four different groups: 268 (41.6%) had completed secondary school, 212 (32.9%) had a bachelor’s degree, 126 (19.5%) had finished elementary school (or less), and only 39 (6.0%) had a master’s degree or higher level of education (Table 1).

**Table 1:** Demographic characteristics of women included in the study

Variable	*n*	%
Residence		
Urban	345	53.5
Rural	300	46.5
Age		
18–25	295	45.7
26–33	187	29.0
34–41	103	16.0
42–49	34	5.3
50–58	22	3.4
> 58	4	0.6
Education		
Elementary or less	126	19.5
Secondary school	268	41.6
Bachelor’s	212	32.9
Master’s or PhD	39	6.0
Total	645	100.0

As seen from Table 2, out of 645 respondents only 0.5% (3) of the women were vaccinated against HPV, whereas 99.5% (642) were not vaccinated.

**Table 2:** Percentage of women ever vaccinated against HPV

Vaccinated	*n*	%
Yes	3	0.5
No	642	99.5
Total	645	100.0

The majority of respondents, 66.4% (or 428 of them) stated that they had no previous knowledge of HPV. One-third of them (215) knew of HPV, and two women (0.3%) did not answer this question. When asked if HPV is a responsible factor for cervical cancer, only 27.6% (or 178) confirmed that they knew this; 7.6% responded “no,” and most of them (64.7% or 417) had no knowledge of this issue. Of the 645 women involved in our study, only 29.6% (or 191) had previously heard of the HPV vaccine, 70.1% had no prior information about the HPV vaccine, and only 0.3% did not answer the question (Table 3).

**Table 3:** Women’s knowledge and awareness of HPV infection, the HPV vaccine, and the connection between HPV, cervical cancer, and STIs

Question	*n*	%
Have you heard about HPV (human papillomavirus)?		
Yes	215	33.3
No	428	66.4
No answer	2	0.3
Is HPV responsible factor for cervical cancer?		
Yes	178	27.6
No	49	7.6
Do not know	417	64.7
No answer	1	0.2
Have you heard about the vaccine against human papillomavirus?		
Yes	191	29.6
No	452	70.1
No answer	2	0.3
Does vaccination against HPV prevent cervical cancer development?		
Yes	155	24.0
No	44	6.8
Do not know	432	67.0
No answer	14	2.2
Have you heard about sexually transmitted infections (STIs)?		
Yes	461	71.5
No	110	17.1
Do not know	73	11.3
No answer	1	0.2
Do you know that STIs can be transmitted among partners?		
Yes	481	74.6
No	35	5.4
Do not know	127	19.7
No answer	2	0.3

The association between several demographic, social, and informational factors and awareness of HPV or knowledge that HPV is related to cervical cancer was studied using multivariable logistic regression, and the results are summarized in Table 4. When controlling for other factors in the regression model, respondents with sexual experience are less likely to have heard about HPV (OR [95% CI] 0.57 [0.32; 0.99]). Women with a university education had more often heard about HPV than women with a elementary education or less (OR [95% CI] 8.33 [3.94; 17.63]), and women that were informed about HPV by nurses (OR [95% CI] 2.75 [1.25; 6.05]), by teachers (OR [95% CI] 2.46 [1.39; 4.34]), or via the internet (OR [95% CI] 1.86 [1.17; 2.94]). Women with a university education were more frequently informed about the relationship between HPV and cervical cancer (OR [95% CI] 6.89 [3.13; 15.18]) compared to those that were informed about the topic by nurses (OR [95% CI] 2.78 [1.28; 6.00]) or/and via the internet (OR [95% CI] 1.9 [1.20; 3.01]). Women that were informed about HPV by friends knew about this relationship less often (OR [95% CI] 0.39 [0.16; 0.97]).

**Table 4:** Factors associated with HPV awareness and knowledge about the relation between HPV and cervical cancer (results of multivariable logistic regression)

	HPV awareness				HPV knowledge			
Factor	No	Yes	aOR (95% CI)	*p*	No	Yes	aOR (95% CI)	*p*
Urban	213 (49.8)	131 (60.9)	1.02 (0.99; 1.05)	0.254	234 (50.2)	110 (61.8)	1.02 (0.98; 1.05)	0.310
Mean age (*SD*)	30.1 (9.3)	26.5 (7.2)	1.01 (0.66; 1.55)	0.962	29.7 (9.3)	26.7 (6.8)	0.99 (0.64; 1.53)	0.947
Sexual experience	369 (86.2)	124 (57.7)	0.57 (0.32; 0.99)	0.048	388 (83.3)	106 (59.6)	0.66 (0.38; 1.17)	0.153
Education								
Elementary (%)	120 (28.0)	13 (6.0)	1		122 (26.2)	11 (6.2)	1	
Secondary (%)	213 (49.8)	46 (21.4)	1.63 (0.80; 3.33)	0.182	221 (47.4)	39 (21.9)	1.72 (0.81; 3.68)	0.161
University (%)	95 (22.2)	156 (72.6)	8.33 (3.94; 17.63)	< 0.001	123 (26.4)	128 (71.9)	6.89 (3.13; 15.18)	< 0.001
Information source								
Doctor (%)	90 (23.6)	69 (36.5)	1.65 (0.95; 2.84)	0.073	99 (23.9)	60 (38.5)	1.57 (0.91; 2.71)	0.107
Nurse (%)	28 (6.5)	31 (14.4)	2.75 (1.25; 6.05)	0.012	31 (6.7)	28 (15.7)	2.78 (1.28; 6.00)	0.010
Parents (%)	14 (3.3)	19 (8.8)	1.97 (0.75; 5.20)	0.169	16 (3.4)	17 (9.6)	1.87 (0.73; 4.77)	0.189
Teachers (%)	37 (8.6)	63 (29.3)	2.46 (1.39; 4.34)	0.002	50 (10.7)	50 (28.1)	1.60 (0.91; 2.79)	0.100
Internet (%)	103 (24.1)	94 (43.7)	1.86 (1.17; 2.94)	0.008	117 (25.1)	80 (44.9)	1.90 (1.20; 3.01)	0.006
Friends (%)	41 (9.6)	18 (8.4)	0.45 (0.19; 1.03)	0.059	46 (9.9)	13 (7.3)	0.39 (0.16; 0.97)	0.043
Media (%)	60 (14.0)	46 (21.4)	0.75 (0.42; 1.36)	0.345	67 (14.4)	39 (21.9)	0.80 (0.45; 1.45)	0.468

The existence of a vaccination against HPV is better known to women with a university education compared to women with an elementary education or less (OR [95% CI] 3.27 [1.65; 6.45]) and compared to those that were informed about vaccination by doctors (OR [95% CI] 1.72 [1.02; 2.88]) or via the internet (OR [95% CI] 2.23 [1.46; 3.42]) (Table 5). That vaccination prevents cervical cancer is better known to women with a university education (OR [95% CI] 3.68 [1.67; 8.13]) and those that were educated about the topic by nurses (OR [95% CI] 3.19 [1.42; 7.17]) or via the internet (OR [95% CI] 2.37 [1.49; 3.78]). Women that received information from friends are less aware of this fact (OR [95% CI] 0.33 [0.12; 0.92]).

**Table 5:** Factors associated with awareness about HPV vaccination and knowledge about the relation between HPV vaccination and cervical cancer (results of multivariable logistic regression)

	HPV vaccination awareness				HPV vaccination knowledge			
Factor	No	Yes	aOR (95% CI)	*p*	No	Yes	aOR (95% CI)	*p*
Urban	230 (50.9)	114 (59.7)	0.99 (0.96; 1.02)	0.509	244 (51.4)	95 (61.3)	1.01 (0.98; 1.05)	0.451
Mean age (*SD*)	29.9 (9.1)	26.5 (7.4)	0.98 (0.66; 1.46)	0.923	29.6 (9.2)	26.5 (7.2)	1.16 (0.73; 1.82)	0.534
Sexual experience	373 (82.5)	121 (63.4)	0.75 (0.44; 1.29)	0.306	390 (82.1)	91 (58.7)	0.64 (0.36; 1.14)	0.129
Education								
Elementary (%)	117 (25.9)	16 (8.4)	1		115 (24.2)	11 (7.1)	1	
Secondary (%)	202 (44.7)	58 (30.4)	1.60 (0.84; 3.03)	0.152	222 (46.7)	35 (22.6)	1.26 (0.58; 2.73)	0.554
University (%)	133 (29.4)	117 (61.3)	3.27 (1.65; 6.45)	< 0.001	138 (29.1)	109 (70.3)	3.68 (1.67; 8.13)	0.001
Information source								
Doctor (%)	96 (24.4)	62 (35.2)	1.72 (1.02; 2.88)	0.040	104 (24.6)	48 (35.8)	1.17 (0.64; 2.12)	0.613
Nurse (%)	33 (7.3)	26 (13.6)	1.76 (0.85; 3.67)	0.129	34 (7.2)	25 (16.1)	3.19 (1.42; 7.17)	0.005
Parents (%)	16 (3.5)	17 (8.9)	2.17 (0.91; 5.22)	0.082	20 (4.2)	13 (8.4)	1.18 (0.45; 3.10)	0.744
Teachers (%)	54 (11.9)	46 (24.1)	1.47 (0.86; 2.50)	0.158	51 (10.7)	47 (30.3)	1.91 (1.08; 3.36)	0.026
Internet (%)	110 (24.3)	87 (45.5)	2.23 (1.46; 3.42)	< 0.001	119 (25.1)	76 (49.0)	2.37 (1.49; 3.78)	< 0.001
Friends (%)	40 (8.8)	19 (9.9)	1.15 (0.57; 2.33)	0.692	45 (9.5)	12 (7.7)	0.33 (0.12; 0.92)	0.035
Media (%)	66 (14.6)	39 (20.4)	0.81 (0.46; 1.41)	0.453	67 (14.1)	38 (24.5)	1.07 (0.60; 1.93)	0.81

## Discussion

To the best of our knowledge, this is the first study of this kind conducted in Kosovo. The overall rate of women ever vaccinated against HPV is extremely low: 0.5%, or only three women out of 645 included in the study. To date, Kosovo has no national programs for HPV vaccination and it has not promoted HPV vaccination. The prevalence of high-risk HPV genotypes among Kosovo women [9] is similar to that in other countries in the region, and the lack of a national screening program for cervical cancer in Kosovo corresponds to the great risk women have of acquiring cervical cancer.

Our results show that women’s knowledge about HPV infection is poor because only 33.3% of women have any information about HPV, and the majority state that they have never heard of HPV. Some similar studies have been conducted worldwide, mainly assessing the knowledge of young adolescents and students, and they yielded different results. Much higher results were obtained in studies in many developed countries [13, 15–17]. In a study of 204 women 16–23 years old attending a public outpatient gynecology clinic in Brazil, Moreira et al. found that 66.7% of women were informed about HPV [18]. However, a study in China showed even lower results, at 19.3% [19].

Most of the women included in the study (72.3%) do not know or think it is not true that HPV is a factor responsible for the development of cervical cancer. Other studies have showed similar results, such as a study in Nigeria [20], whereas a survey in Australia revealed that 66% of men and women know about HPV and cervical cancer [21].

Two-thirds of Kosovo women stated that they had never heard about the HPV vaccine, and only 29.6% of them have some information about the HPV vaccine. The percentage of women that know that the HPV vaccine can prevent the development of cervical cancer is even lower (24%). These results are far lower than the rates reported in studies conducted in other countries [17, 18, 21, 22]. The results of this study also confirm previous findings by other authors that a higher education level and younger age have an impact on the level of knowledge and awareness of HPV infection, vaccination, and its relation to cervical cancer [22, 23].

Women with higher education and younger in age, have more access to the proper information, as a result of easier access to updated information from the media, internet or literature, increased awareness and regular consultations with doctors, therefore they are more keen to understand the importance of HPV infection and its relationship to cervical cancer, as well as HPV vaccination as the most important measure to prevent cervical cancer.

This study has some limitations. As a cross-sectional study, it may exclude causal relationships between various factors and results. Also, because the information was collected through questionnaires, there is a possibility of incorrect answers, which could lead to possible bias. Despite these possible limitations, the study had quite a high rate of response. Information was gathered from a large number of participants, making it a very representative sample.

## Conclusion

Based on our results, the level of knowledge and awareness of HPV infection, the HPV vaccine, and its causal association with cervical cancer is generally very low among Kosovo women. Also, it can be concluded that the level of vaccination against HPV in Kosovo is extremely low. The results also indicate that having a higher level of education, living in urban areas, and being younger are favorable factors for having more information about HPV infection and the HPV vaccine.

Based on our study data, there is an immediate need for the development of a proper educational program about HPV infection and the importance of HPV vaccination as a preventative measure against the development of cervical cancer.

## Supplementary Information

**Additional file 1**. Questionnaire.

## Data Availability

The datasets used and/or analysed during the current study are available from the corresponding author on reasonable request.
