# Supplementary material for: Kosovo women’s knowledge and awareness of human papillomavirus (HPV) infection, HPV vaccination, and its relation to cervical cancer
Source: BMC Womens Health. 2021 Oct 9;21:354. doi: 10.1186/s12905-021-01496-x (PMC8502331; doi:10.1186/s12905-021-01496-x)
Supplement: Supplementary file 1 — Additional file 1. Questionnaire. [file 12905_2021_1496_MOESM1_ESM.docx]

**Kosovo women’s knowledge and awareness of human papillomavirus (HPV) infection, HPV vaccination, and its relation to cervical cancer**

Research design

Our research is designed to explore the knowledge, behavior and perception of Kosovo women regarding Human papillomavirus (HPV) infection, HPV vaccination and cervical cancer.

The research will include 800 women in the age group of 18 to 60 years.

This is anonym self-filled questionnaire and data from this study will be used only for publication purposes.

*Please answer the following questions honestly.*

*Circle a correct answer.*

1) Your age?_______

2) Where do you live?

a. Urban area (City)

b. Rural area (Village)

3) What is your level of education?

a. les then elementary school

b. completed elementary school

c. secondary school

d. professional school

e. bachelor

f. master or PhD

4) Have you had sexual intercourse?

a. Yes

b. No

5) What was the age at which you had your first sexual contact?________________

6) Have you heard of sexually transmitted diseases?

a. Yes

b. No

c. Do not know

7) Women and their partner can be infected, and can carry the infection to each other?

a. Yes

b. No

c. Do not know

8) Have you heard of Human Papillomavirus (HPV)?

a. Yes

b. No

9) Human Papillomavirus is responsible for cervical cancer?

a. Yes

b. No

c. Do not know

10) Have you heard of the vaccine which protects women from HPV infectrion?

a. Yes

b. No

11) Have you ever been vaccinated with HPV vaccine?

a. Yes

b. No

12) Preventing HPV infection prevents cervical cancer?

a. Yes

b. No

c. Do not know

13) Have you ever heard of the PAP test?

a. Yes

b. No

14) Do you know what the PAP test is for?

a. Yes

b. No

15) Have you ever had PAP test done?

a. Yes

b. No

16) If you did the PAP test, at what time intervals did you repeat it?

a. Only once

b. Every year

c. Every 3 years

d. Do not consider necessary to repeat it

17) have you ever recived information regardin cervical cancer?

a. Yes

b. No

18) What has been the source of information on sexually transmitted diseases, HPV and PAP testing and cervical cancer?

a. Doctor and Nurse

b. Teachers at school

c. Parents

d. Friends

f. Visual and written media

g. Internet sources

h. Other sources

_________________________________________________________________________

***Thank you for your time and sincerity!***

***This research will help educate and improve women’s health.***
